# Genomic Analysis Uncovers Immune Microenvironment Characteristics and Drug Sensitivity of Ferroptosis in Breast Cancer Brain Metastasis

**DOI:** 10.3389/fgene.2021.819632

**Published:** 2022-01-25

**Authors:** Lei Zhu, Mu Chen, Bingsong Huang, Tao Zhang, Kui Chen, Hao Lian, Min Liu, Kaijun Zhao, Ying Pang, Jing Zhang, Qinchuan Li, Chunlong Zhong

**Affiliations:** ^1^ Department of Neurosurgery, Shanghai East Hospital, School of Medicine, Tongji University, Shanghai, China; ^2^ Department of Thoracic Surgery, Shanghai East Hospital, School of Medicine, Tongji University, Shanghai, China; ^3^ Institute for Advanced Study, Tongji University, Shanghai, China

**Keywords:** ferroptosis, breast cancer, brain metastasis, prognosis, drug sensitivity

## Abstract

**Background:** The role of ferroptosis in breast cancer brain metastasis (BCBM) is unclear. This study aimed to explore the ferroptosis-related genes (FRG) relations with the tumor microenvironment, as well as evaluate their values in predicting survival and drug sensitivity in patients with BCBM.

**Materials and Methods:** Genes expression and clinical data were downloaded from Gene Expression Omnibus (GEO). Univariate and multivariate Cox regression analyses were performed to explore the independent prognostic factors. Consensus cluster principal component analysis (PCA) was used to establish the ferroptosis score. Immunological signatures were analyzed by the single-sample gene set enrichment analysis (ssGSEA). Drug sensitivity was evaluated through the estimated half-maximal inhibitory concentration (IC50). Finally, results were validated in external cohorts.

**Results:** Fourteen significantly different FRG were identified between breast cancer (BC) and BCBM tissues. Survival analysis demonstrated HMOX1, PEBP1, KEAP1, and LPCAT3 were significantly associated with overall survival (OS) and relapse-free survival (RFS) (all *p* < 0.05). High ferroptosis score was correlated with iron ion homeostasis, iron metabolism, higher stromal cells and immune cells scores. Patients with high- and low-ferroptosis scores were characterized by different drug sensitivities. Following external validations, the ferroptosis had distinct expression profiles between the BC and BCBM, and could serve as biomarkers for OS and drug response.

**Conclusion:** Our findings suggested that ferroptosis may be involved in the process of BCBM, and ferroptosis could serve as prognostic biomarkers. Evaluation of ferroptosis may deepen our understanding about the tumor microenvironment, and could help clinicians to make individualized therapy.

## Introduction

Breast cancer (BC) is the most prevalent tumor in women worldwide, ranking the third most common malignancy followed by lung and colon cancer. It’s reported that approximately 1,700,000 new cases and almost 500,000 deaths per year globally (Ferlay et al., 2015; Torre et al., 2015). Breast cancer brain metastasis (BCBM) becomes a major limitation of life expectancy and remains a substantial contributor to overall mortality. Nearly 5–20% breast cancer will develop brain metastasis, and it is the second common primary tumor associated with brain metastasis after lung cancer (Achrol et al., 2019). Breast cancer patients with basal-like (25–27%) and HER2-enriched cancer (11–20%) have higher propensities to metastasize to the brain, compared with those in luminal A (8–15%) and luminal B (11%) subtypes (Kennecke et al., 2010). Since no clinically approved biomarkers of brain metastasis is available, and the presences or absences of estrogen receptor (ER), progesterone receptor (PgR), HER2 and Ki67 status are not sufficient to accurately prognosticate metastasis due to heterogeneity between primary and metastatic sites (Harbeck and Gnant, 2017). Hence, BCBM is often diagnosed late and represents dismal survival.

Diverse underlying mechanisms such as gene alterations, immune dysregulation, as well as estrogen and progesterone imbalance have resulted in the poor prognosis (Shiovitz and Korde, 2015; Bates et al., 2018; Vaz-Luis and Partridge, 2018; Terry et al., 2019). It’s estimated that the 1-year survival rate of patients with BCBM is merely 20%, although the tremendous progress has been made in the multidisciplinary treatment, including surgery, chemoradiotherapy and endocrine therapy (Arslan et al., 2010). Considering the therapeutic resistance, several targeted agents such as lapatinib, pazopanib have been tested in patients with BCBM. However, the clinical trial failed to demonstrate improved survival, except for a small subset of patients (Seligmann et al., 2020). Therefore, it’s imperative to identify biomarkers that could optimize the implementation of precision targeted therapy.

A thorough understanding of molecular mechanisms that drive BCBM should aid in the discovery of novel strategies to improve clinical management. The extensive applications of high-throughput sequencing technologies in cancer biology, such as cell death analysis, have revealed the relations between thousands of aberrant gene expressions associated with BCBM patients. There is increasing evidence showing that ferroptosis, an iron-catalyzed form of regulated necrosis, plays important role in various cancers, including breast tumors, brain tumors and breast cancer metastasis (Ma et al., 2016; Nagpal et al., 2019; Weiland et al., 2019).

Ferroptosis can be induced by iron accumulation, glutathione (GSH) depletion, glutathione peroxidase 4 (GPX4) inactivation, and is characterized by lipid peroxidation products and toxic reactive oxygen species (ROS) derived from iron metabolism (Stockwell et al., 2017; Bersuker et al., 2019). Several studies have shown that ferroptosis could be triggered in BC (Ma et al., 2016; Yu et al., 2019a). The activities of ferroptosis are regulated precisely by ferroptosis-related genes (FRG), and its dysfunction links with many kinds of diseases (Stockwell et al., 2017; Bersuker et al., 2019; Weiland et al., 2019). In addition, the activation of ferroptosis has tumor suppression efficacy and exerts great potential as a salient anti-cancer target. However, the role of ferroptosis in BCBM is unclear, and few studies explored the relationship between ferroptosis and survival in patients with BC. In this study, we aimed to investigate and validate the FRG signatures that correlate with BCBM, as well as evaluate the FRG values in predicting prognosis and drug sensitivity.

## Materials and Methods

### Data Collection and Extraction

Gene expression data and clinical information of patients with BCBM were downloaded from Gene Expression Omnibus (GEO) database (https://www.ncbi.nlm.nih.gov/gds/). Two datasets (GSE10893 and GSE43837) were used in our study. Both experiment types were expression profiling by array, and both of them contained primary breast cancer and breast cancer brain metastatic tissues. The raw data of gene expressions were selected by “GEO2R” online tool and normalized using the “limma” package in R software (version 4.0.3). The 60 FRG were retrieved from previously published literatures and were available in the Supplementary Table S1 (Stockwell et al., 2017; Bersuker et al., 2019; Hassannia et al., 2019).

### Identification of Significantly Different Genes and Enrichment Analysis

The significantly different genes (SDG) among the FRG were identified using “GEO2R” and the “limma” package with the Wilcoxon test. The cut-off value was determined with *p* < 0.05. SDG interactions were performed through “igraph” “corrplot” packages in R software. To get more reliable data, the overlapping FRG between GSE10893 and GSE43837 were identified and were used for further analysis.

Gene Ontology (GO), including the biological process (BP), cellular component (CC) and molecular function (MF), was performed by “clusterProfiler” package in R software. The Kyoto Encyclopedia of Genes and Genomes (KEGG) was also done using the same tool.

Functional similarity refers to semantic correlation and biological resemblance, which could be used for the purpose of assessing the intimacy and relationship between each gene and its partners by evaluating function and location. Functional similarity was performed by the “GOSemSim” R package (Wang et al., 2007).

### Prognostic Survival Analysis

GSE10893 contained gene expression data and corresponding clinical information, and thus was used to perform survival analysis. Univariate and multivariate Cox regressions were used to assess the relationships between the SDG and the patients’ overall survival (OS) and relapse-free survival (RFS). In order to explore the independent risk factors of OS and RFS, we combined the FRG with clinical information using the univariate Cox regression. Significant prognostic factors (*p* < 0.05) were then enrolled into multivariate Cox regression. Independent prognostic genes were used to calculate the risk score following formula: risk score = ${\sum^{\text{​}}}_{n=1}^{j}\;Coefj\;*\;Xj$ , with Coef j representing the coefficient and Xj representing the relative expression levels of each SDG standardized by z-score. Patients were divided into high- and low-risk groups according to the median of the risk score. Furthermore, we analyzed the correlations between the SDG and clinical features using the *t*-test or Kruskal-Wallis test.

### Single-Sample Gene Set Enrichment Analysis

The infiltrating score of 16 immune cells and the activity of 13 immune-related pathways were calculated with single-sample gene set enrichment analysis (ssGSEA) by the “gsva” package in R (Rooney et al., 2015). The effects of FRG on immune cells were assessed using linear regression. The annotated gene set file is provided in Supplementary Table S2.

### Establishment of Ferroptosis Gene Signature

The unsupervised clustering was executed by the “ConsensuClusterPlus” R package, and the classification was confirmed by 1000 times permutations. Principal component analysis (PCA) was implemented to classify the patients with BC into groups A and B. Then, the ferroptosis score was defined according to the similar method by a previous study: ferroptosis score = $\sum^{\text{​}}\text{group A}$ - $\sum^{\text{​}}\text{group B }$ (Zhang et al., 2020). Where the group A represented the first component in the PCA, and group B  presented the second component in the PCA.

### Tumor Microenvironment and Drug Sensitivity Analysis

To explore the effects of ferroptosis on stromal and immune cells in TME, we calculated the stromal cells and immune cells score by applying the Estimation of STromal and Immune cells in MAlignant Tumour tissues using Expression data (ESTIMATE) algorithm (Yoshihara et al., 2013). We compared the stromal cells, immune cells and tumor purity differences between the high- and low-ferroptosis score groups.

Genomics of Drug Sensitivity in Cancer (GSDC) is the largest public resource for cancer cell drug sensitivity and drug response molecular marker, containing 1000 human cancer cell lines and 100s of compounds (https://www.cancerrxgene.org/). On this website, you will find drug response data and genomic markers of sensitivity. To identify the molecular characteristics related to drug sensitivity and resistance, we applied the database to predict the targeted and chemotherapeutic responses by estimating the half-maximal inhibitory concentration (IC50) (Yang et al., 2013). The IC50 of each patient was estimated by the pRRophetic R package (Geeleher et al., 2014). Lastly, the IC50 differences were calculated between the high- and low-ferroptosis score groups.

### Potential Small Molecular Compounds Prediction

To screen potential drugs that could target BC, we conducted the small molecular compounds analysis in the Connectivity map (CMap; http://portals.broadinstitute.org/cmap/) database. Firstly, we compared the SDG between high- and low-ferroptosis score groups. Then, we investigated the SDG functions through GO and KEGG enrichment analyses. Lastly, the up- and down-regulated genes were uploaded into the CMap website, and potential drugs and mechanisms were predicted.

### External Validation

**FIGURE 1:**
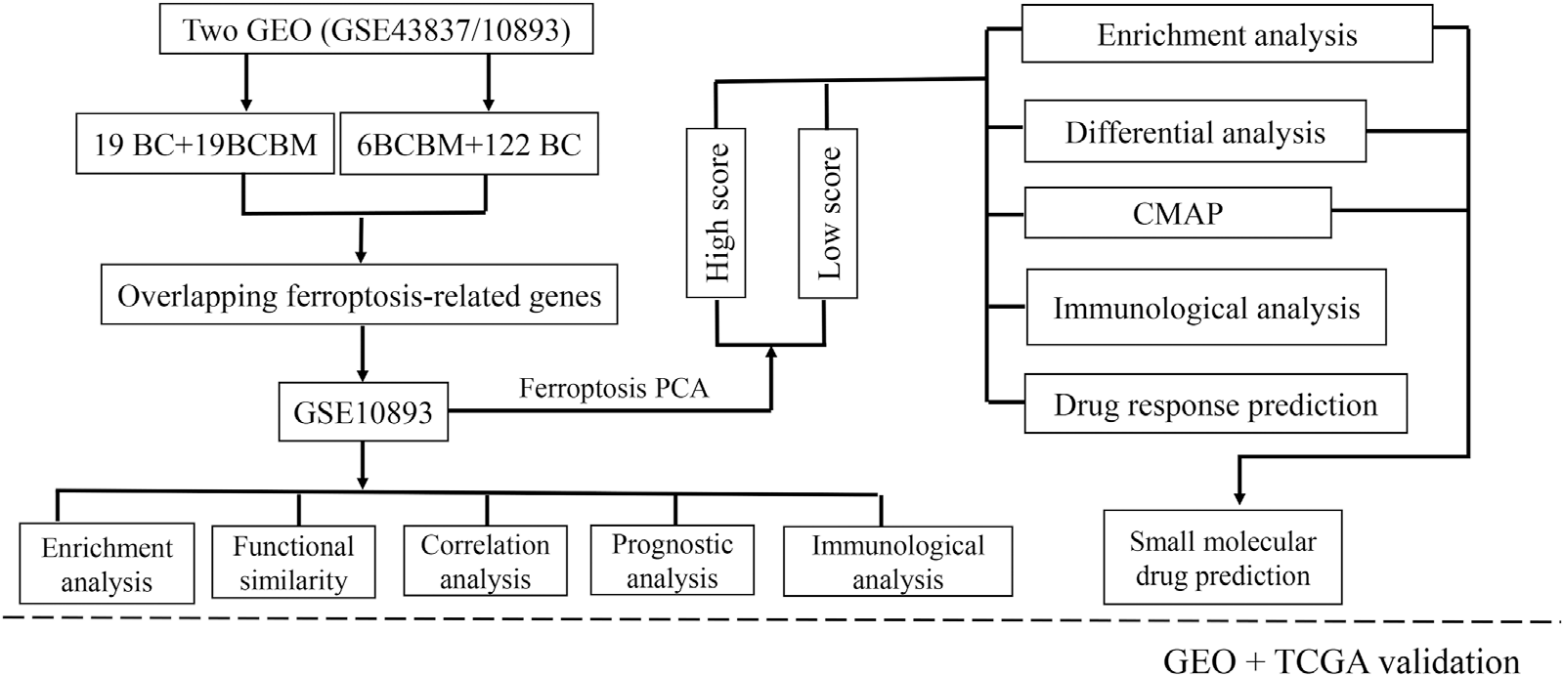
Flowchart of the study design. BCBM: breast cancer brain metastasis; BC: breast cancer; GO: Gene Ontology; KEGG: Kyoto Encyclopedia of Genes and Genomes; PPI: protein-protein interaction; CMAP: Connectivity Map; GEO: Gene Expression Omnibus; TCGA: the Cancer Genome Atlas.

We used another dataset from GEO (GSE125989) to verify the FRG, which contained 16 primary BC and 16 matched BCBM tissues. The primary goal was to verify the expression profiles of FRG between BC and BCBM tissues, and explore the ferroptosis-related cluster patterns and relations with TME. The second goal was to investigate the prognostic values of ferroptosis in BC patients with and without metastasis. The prognostic values of ferroptosis were validated in The Cancer Genome Atlas (TCGA) dataset by performing Kaplan-Meier survival analysis. In addition, the drug sensitivity was also assessed in patients from TCGA. The gene expression data in GSE125989 were provided in Supplementary Table S3, and TCGA data can be obtained online (https://portal.gdc.cancer.gov/). The overall design of this study was seen in Figure 1.

### Statistical Analysis

Wilcoxon test was used to compare gene expression differences between BC and BCBM tissues. Univariate and multivariate Cox regression analyses were used to evaluate the correlation between the genes and OS, RFS. Log-rank test was used to compare the survival differences, and Kaplan-Meier curves were implemented to visualize the survival. Mann-Whitney test with *p* values adjusted by the BH method was used to compare the ssGSEA scores of immune cells or pathways between the two groups. Spearman correlation analysis was used to evaluate the interactions. All the statistical analyses were done using the R software (version 4.0.3). *p* < 0.05 was set as statistically significant.

## Results

GSE10893 contained 275 samples from primary breast cancer tissues and 35 metastatic tissues measured by microarray, and they were sequenced on different platforms. To reduce the heterogeneity and we selected the samples on the GPL1390 (*n* = 185). We exclude eight normal samples, and 49 samples without clinical information. A total of 128 samples with gene expression profiles and clinical information were downloaded from the GSE10893, including 6 BCBM samples and 122 BC samples. GSE43837 contained 19 BC and matched 19 BCBM tissues, and 57 FRG were obtained from this dataset (Supplementary Table S4). A total of 14 overlapping FRG were identified. Among them, 7 genes were significantly upregulated in the BCBM compared with BC tissues, and 7 genes were significantly downregulated (Table 1).

**TABLE 1:** Significantly differentially expressed FRG in BC and BCBM tissues.

Gene	logFC	t	B	FDR	*p* Value
DPP4	−1.824	−3.753	0.317	0.039	0.000
HMOX1	−1.130	−2.251	−3.608	0.330	0.026
GLS2	−0.911	−3.063	−1.696	0.208	0.003
CD44	−0.677	−2.276	−3.558	0.324	0.025
ACO1	−0.663	−2.384	−3.331	0.295	0.019
ALOX5	−0.658	−2.293	−3.521	0.320	0.023
KEAP1	−0.476	−2.495	−3.089	0.257	0.014
LPCAT3	0.371	2.220	−3.666	0.301	0.020
G6PD	0.437	2.091	−3.922	0.386	0.038
CS	0.459	2.759	−2.473	0.189	0.007
PEBP1	0.509	2.303	−3.501	0.317	0.023
FDFT1	0.638	2.406	−3.282	0.288	0.018
HSBP1	0.742	2.715	−2.579	0.200	0.008
TFRC	0.977	2.545	−2.976	0.191	0.007

BC: breast cancer; BCBM: breast cancer brain metastasis; LogFC: log fold change; FDR: false discovery rate; t: *t*-test statistics; B: regression coefficient.

### Gene Interaction Network and Enrichment Analysis

**FIGURE 2:**
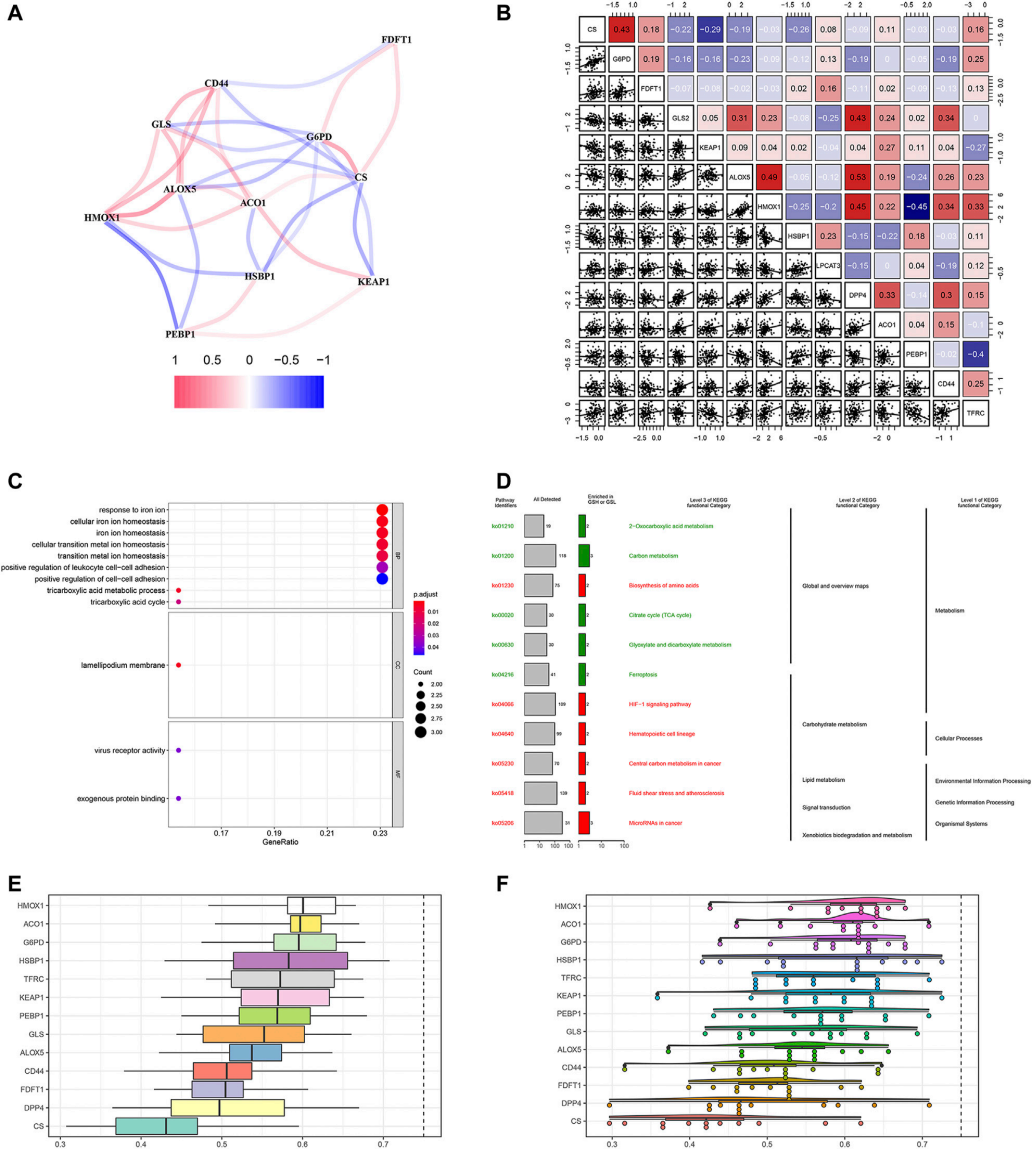
Gene correlation network and enrichment analysis. **(A)** The correlation network of FRG. The correlation coefficients are represented by different colors, in which red lines represent positive correlation, while negative in blue. **(B)** Gene interaction analysis. The correlations were represented in the data. **(C)** GO analysis results showed FRG were enriched in the iron homeostasis and ferroptosis-related pathways. **(D)** KEGG enrichment demonstrated metabolic activities and ferroptosis. **(E)** Summary of FRG similarities. The boxes indicated the middle 50% of the similarities; and the upper and lower boundaries show the 75th and 25th percentile. **(F)** Raincloud plots of OGG. Data were shown as the mean and standard error. Each dot represented a single gene. The dashed line represents the cutoff value (0.75).

We firstly investigated the 14 FRG interactions through the “corrplot” R package (Figure 2A). The results showed ALOX5 and DDP4 had the strongest positive correlation (*r* = 0.53), implying they were synergetic contributors to the genetic architecture of ferroptosis, and resulted in the susceptibility of breast cancer. While the HMOX1 and PEBP1 had the strongest negative correlation (*r* = −0.45), implying they exerted the opposite effects on the ferroptosis (Figure 2B).

Then, we performed the GO enrichment analysis using the 1660 SDG, which showed the SDG were enriched in the iron homeostasis and iron-related transition pathways. In the BP process, they were strongly associated with iron ion homeostasis. In the CC and MF processes, the SDG were enriched in the lamellipodium membrane and virus receptor activity (Figure 2C). KEGG enrichment analysis showed that these SDG were significantly enriched in the ferroptosis and metabolic activities (Figure 2D).

Based on the GO analysis and semantic similarities, we ranked the FRG by average functional similarities between every gene and their partners, with the cut-off value of 0.75. The box plots and raincloud plots were demonstrated in Figures 2E,F. The results implied the HMOX1 and ACO1 had significant similarities, suggesting they functioned consistently.

### Prognostic FRG for OS and RFS

To investigate the prognostic values of FRG, we performed survival analysis. We found that HMOX1 and PEBP1 were significantly associated with OS (*p* = 0.035, 0.017 respectively). However, they were not significantly statistical in the multivariate Cox regression analysis (*p* > 0.05). For RFS, the KEAP1 and LPCAT3 had significant prognostic values in the multivariate cox regression (*p* = 0.002, 0.008 respectively) (Table 2).

**TABLE 2:** cox regression analysis of OS and RFS in breast cancer patients.

Gene	Univariate cox regression			Multivariate cox regression		
	HR	95%CI	P	HR	95%CI	*P*
HMOX1^a^	2.100	1.054–4.186 0.035	1.788	0.906–3.526	0.094	—
PEBP1^a^	0.421	0.207–0.858	0.017	0.522	0.241–1.130	0.100
KEAP1^b^	0.722	0.595–0.875	<0.001	0.745	0.616–0.900	0.002
LPCAT3^b^	2.152	1.106–4.187	0.024	2.536	1.281–5.018	0.008

HR: hazard ratio; CI: confidence interval.

a Genes related OS.

b Genes related RFS.

**FIGURE 3:**
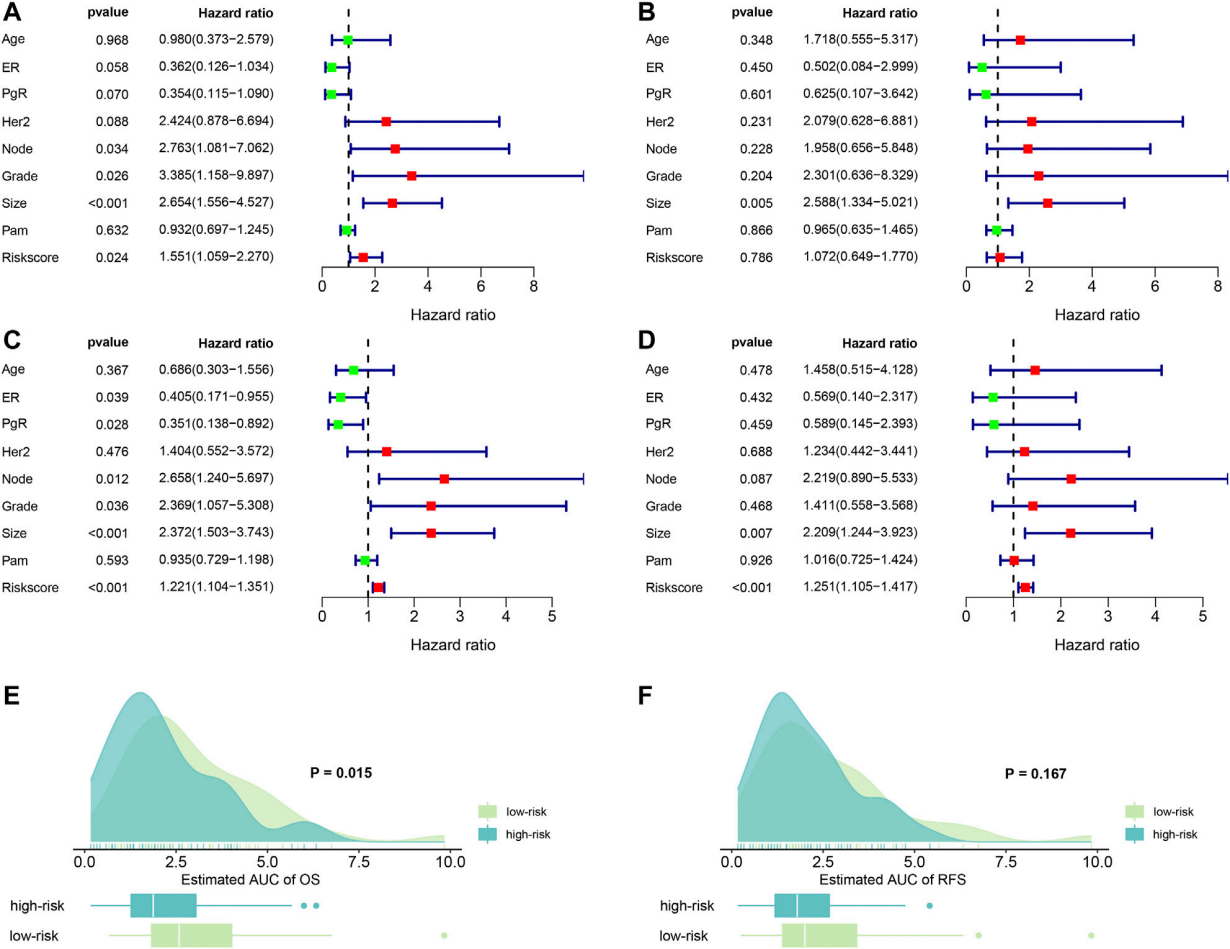
Forest plots of independent risk factors and survival prediction. **(A)** univariate Cox regression analysis for OS. **(B)** Multivariate Cox regression analysis for OS. **(C)** Univariate Cox regression analysis for RFS. **(D)** multivariate Cox regression analysis for RFS. **(E)** OS prediction of the estimated AUC using the risk score. **(F)** RFS prediction of the estimated AUC using the risk score. ER: estrogen receptor; PgR: progesterone receptor; node: lymph node number; Grade: tumor grade; size: tumor size; PAM: a classification method.

According the median of the risk score (risk score = ${\sum^{\text{​}}}_{n=1}^{j}\;Coefj\;*\;Xj$ , risk score for OS = 0.581 * expression level of HMOX1 + −0.650 * expression level of PEBP1; risk score for RFS = −0.295 * expression level of KEAP1 + 0.930 * expression level of LPCAT3), patients were stratified into high- and low-risk groups respectively. Then, we combined the clinical information with gene expression levels to explore the independent risk factors. In the univariate cox analysis, we found that lymph node number, tumor grade, tumor size and risk score were significantly associated with OS (all *p* < 0.05) (Figure 3A). Moreover, the multivariate cox regression showed the tumor size was the only independent risk factor for OS (HR = 2.588, *p* = 0.005) (Figure 3B). For RFS, the ER, PgR status, lymph node number, tumor grade, tumor size and risk score were significantly associated with RFS (all *p* < 0.05) (Figure 3C). And the multivariate cox regression results showed that tumor size and risk score were independent risk factors for RFS in breast cancer patients (HR = 2.209, *p* = 0.007; HR = 1.251, *p* < 0.001 respectively) (Figure 3D).

Next, we examined the predictive abilities using the risk score. By evaluating the area under curves (AUC), we found that risk score had excellent ability to discriminate the OS between the high- and low-risk groups (*p* = 0.015). Similarly, we found that risk score also demonstrated good predictive abilities to predict RFS. However, the difference was not statistical (*p* = 0.167). The results are visualized in Figures 3E,F.

### Prognostic Hazard Curves in High- and Low-Risk Patients

**FIGURE 4:**
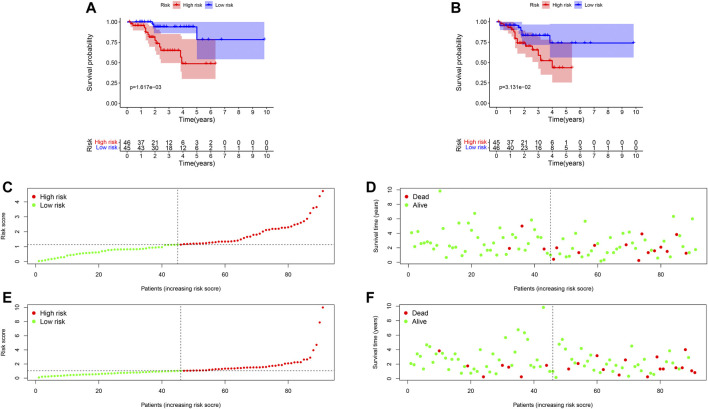
Survival comparison and risk score hazard analyses of high- and low-risk groups in breast cancer patients. **(A)** K-M curve for OS; The result showed the survival status is significantly different between the two groups (*p* = 0.004). **(B)** K-M curve for RFS; High-risk groups patients had worse RFS than that in the low-risk group (*p* = 0.021). **(C)** Risk score hazard curve for OS. The dotted line indicated the individual inflection point of the risk score curve, by which the patients were categorized into low- and high-risk groups. Red represented high-risk and green represented low-risk. **(D)** Risk score scatters plot of OS in high and low-risk groups. Red dots represented the dead patients and green represented the alive. With the increase of risk scores, more patients died. **(E)** Risk score hazard curve for RFS. **(F)** Scatter plot of patients for RFS.

Ninety-one primary breast cancer patients were divided into high- and low-risk patients (*n* = 46, 45, respectively) according to the median of the risk score (Thirty-six patients’ information were incomplete and one brain metastasis patient was excluded). The Kaplan-Meier (K-M) curve showed that patients with high-risk score had a significant higher death probability than those with low-risk (median time = 1.333 vs. 3.417 years, *p* = 0.002) for OS (Figure 4A). Similarly, patients in high-risk score group had worse RFS than those with low-risk scores (median time = 1.250 vs. 3.083 years, *p* = 0.031) (Figure 4B). In addition, we performed prognostic hazard analysis between high- and low-risk score groups. The results showed that as the risk score increased, the patients’ death risk increased, and the survival time decreased (Figures 4C–F).

### Ferroptosis Is Correlated With Clinical Characteristics

**FIGURE 5:**
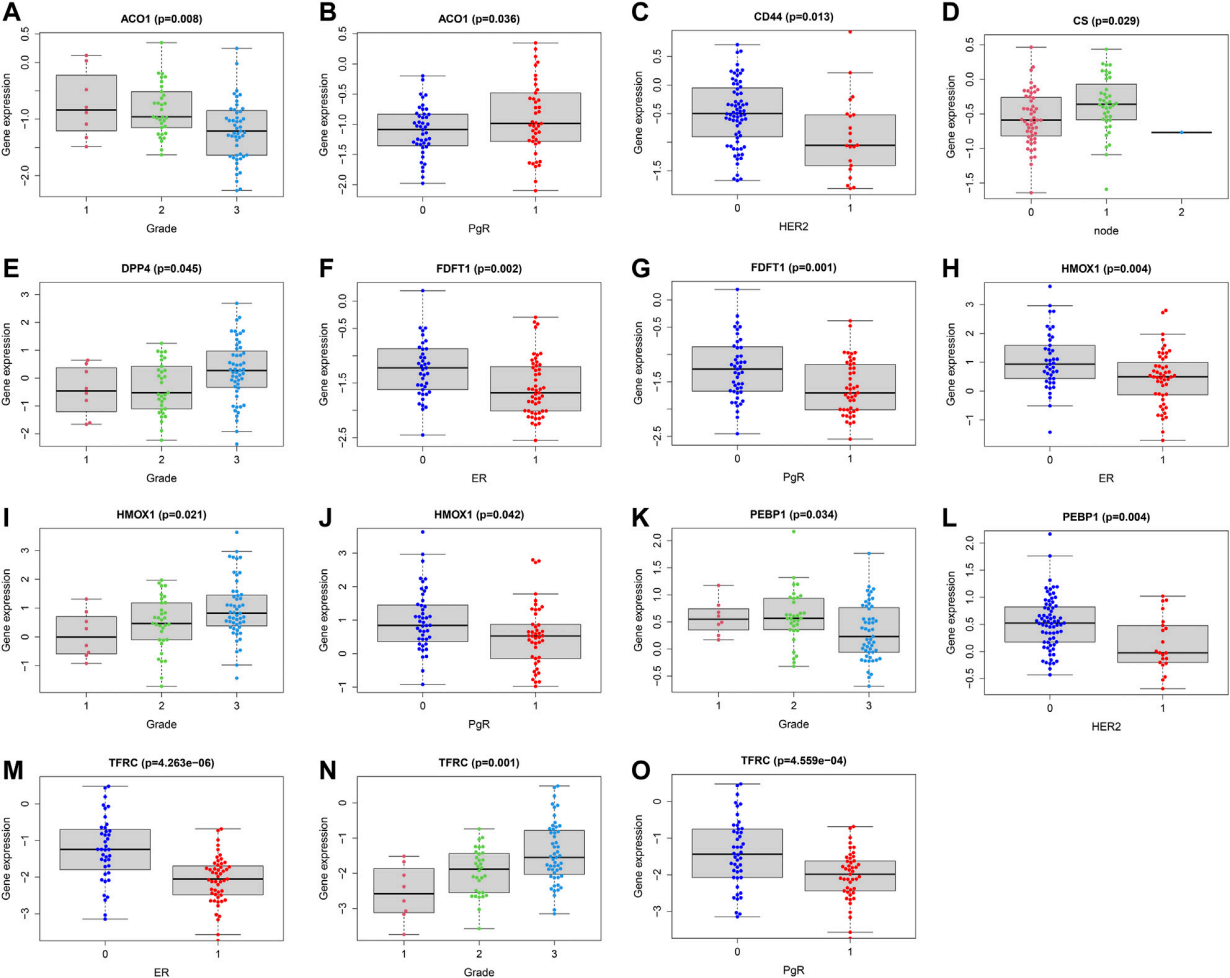
Correlations analysis between FRG and patients’ clinical characteristics. Correlations between ACO1 and tumor grade **(A)** and PgR **(B)**. Correlations between CD44 and HER2 **(C)**. Correlations between CS and lymph nodes **(D)**. Correlations between DPP4 and tumor grade **(E)**. Correlations between FDFT1 and ER **(F)** and PgR **(G)**. Correlations between HMOX1 and ER **(H)**, tumor grade **(I)** and PgR **(J)**. Correlations between PEBP1 and tumor grade **(K)** and HER2 **(L)**. Correlations between TFRC and ER **(M)**, tumor grade **(N)** and PgR **(O)**. ER (1 = positive; 0 = negative); PgR (1 = positive; 0 = negative); HER2 (1 = positive; 0 = negative); node status (1 = positive; 1 = 2 or more nodes+; 0 = negative); size (1 = ≤2 cm; 2 = 2-5 cm; 3 = >5 cm; 4 = any size with direct extension to chest wall or skin).

In order to assess whether ferroptosis was correlated with patients’ clinical information, we calculated statistical differences between the 14 FRG and clinical features using the *t*-test or Kruskal-Wallis test. The results demonstrated that ACO1, DPP4, HMOX1, and TFRC were significantly associated with breast cancer grades (all *p* < 0.05). As the tumor grade increased, DPP4, HMOX1, and TFRC expression levels also increased. The results suggested these genes may be significant contributors to tumor grade, and inhibiting their expressions may control the increase of tumor grade. While, the ACO1 was the opposite. The result suggested ACO1 was a negative contributor of tumor grade, and increasing the ACO1 expression may play the protective role of tumor grade. The ACO1, FDFT1, DPP4, HMOX1, and TFRC were closely associated with patients’ ER and PgR status (all *p* < 0.05). Moreover, we also found CD44 and PEBP1 were significantly correlated with Her2 status in patients with BC (all *p* < 0.05). The details were shown in Figures 5A–O.

### Effects of Ferroptosis on Breast Cancer Subtypes

To further evaluate the effects of ferroptosis on breast cancer subtypes, we systematically investigated the relations between breast cancer subtypes and ferroptosis, and compared the FRG expression profiles differences. According to the Her2, ER, and PgR expressions, we divided patients into HER2-enriched, triple-negative breast cancer (TNBC) and other subtypes (*n* = 8, 28, 55, respectively). The FRG expression levels of the three subtypes were shown in Supplementary Figure S1. Next, we investigated the prognostic values of FRG in the TNBC and other subtypes (HER2-enriched subtype was not performed survival analysis due to the small sample number.). The results showed that ACACA (HR = 7.917, *p* = 0.041) and DPP4 (HR = 0.535, *p* = 0.040) were independent prognostic factors for OS in patients with HER2-enriched subtype. GSS (HR = 0.541, *p* = 0.046) and HMOX1 (HR = 2.184, *p* = 0.031) were independent prognostic factors for OS in patients with other subtypes (Supplementary Figure S2).

Then, we calculated the correlations between FRG and clinical features in patients with the three subtypes. For HER2-enriched subtype, KEAP1 was significantly associated with breast cancer grade and lymph node metastasis (all *p* < 0.05) (Supplementary Figure S3). For TNBC, CD44, FDFT1, G6PD, and GLS2 were significantly associated with breast cancer grade (all *p* < 0.05). G6PD was significantly associated with breast cancer grade lymph node metastasis (*p* = 0.021) (Supplementary Figure S4). For other subtypes, CS and PEBP1 were significantly associated with breast cancer size (all *p* < 0.05). GLS2 and PEBP1 were significantly associated with breast cancer grade (all *p* < 0.05) (Supplementary Figure S5).

### Ferroptosis and Immune Status

**FIGURE 6:**
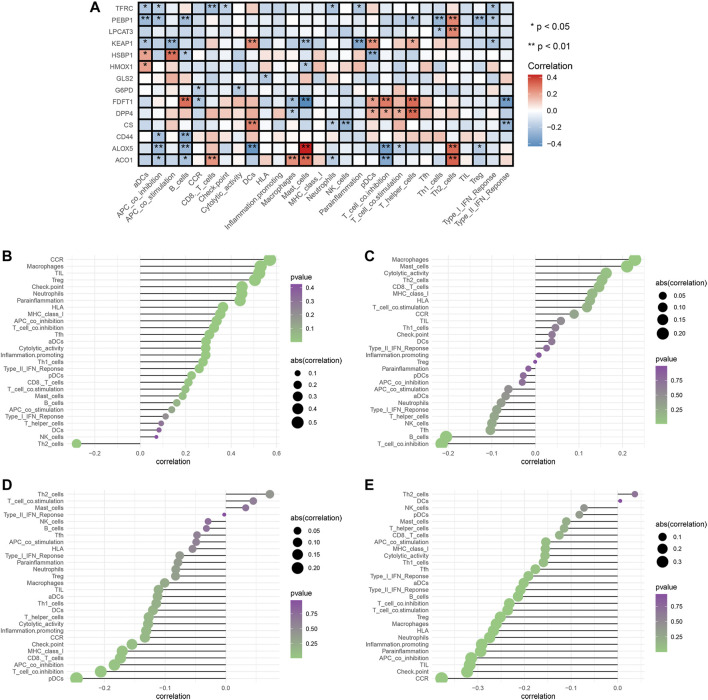
Analysis of ferroptosis-related genes effects on immune signatures. **(A)** Heatmap was demonstrating the correlation between 14 genes and the ssGSEA scores of 29 immune signatures. **(B)** HMOX1 and immune signatures. **(C)** KEAP1 and immune signatures. **(D)** LPCAT3 and immune signatures. **(E)** PEBP1 and immune signatures. Spearman correlation analysis was used to evaluate the relations with *p* < 0.05. *p < 0.05; **p < 0.01. DCs: dendritic cells, iDCs: immature DCs, pDCs: plasmacytoid dendritic cells, TIL: tumor infiltrating lymphocyte, CCR: cytokine-cytokine receptor, APC: antigen presenting cells.

To explore the relationships between FRG and immune cells and immune functions, we quantified the 16 immune cell subtypes and 13 immune functions by “ssGSEA” package in R software. We found the FRG had significant effects on the content of immune cells and immunological functions, especially the aDCs, B cells, iDCs, neutrophils, Tfh, Th2, TIL (all *p* < 0.05) (Figure 6A). Additionally, the antigen presentation process, including APC co-inhibition, APC co-stimulation, HLA, IFN response were significantly influenced by different FRG (all *p* < 0.05).

The tumor microenvironments (including immune cells) are responsible for tumor metastasis and patients’ survival (Arslan et al., 2010; Bates et al., 2018). To fully explore relationships between FRG and immune, we selected the four prognostic genes (HMOX1, PEBP1, KEAP1, LPCAT3) and performed the Spearman correlation analysis by “limma” package. The results showed the HMOX1 was strongly associated with Th2 cells, and chemokine receptors (CCR) (all *p* < 0.05) (Figure 6B). KEAP1 had a close relationship with macrophages and T cell co-inhibition (all *p* < 0.05) (Figure 6C). LPCAT3 showed close relationships with Th2 cells and pDCs (all *p* < 0.05) (Figure 6D). PEBP1 exhibited significant associations with Th2 cells and chemokine receptors (CCR) (all *p* < 0.05) (Figure 6E). Collectively, these findings suggested that FRG were accompanied by changes in immune cell contents and immunological functions.

### Ferroptosis-Related Cluster Patterns and Relations With TME

**FIGURE 7:**
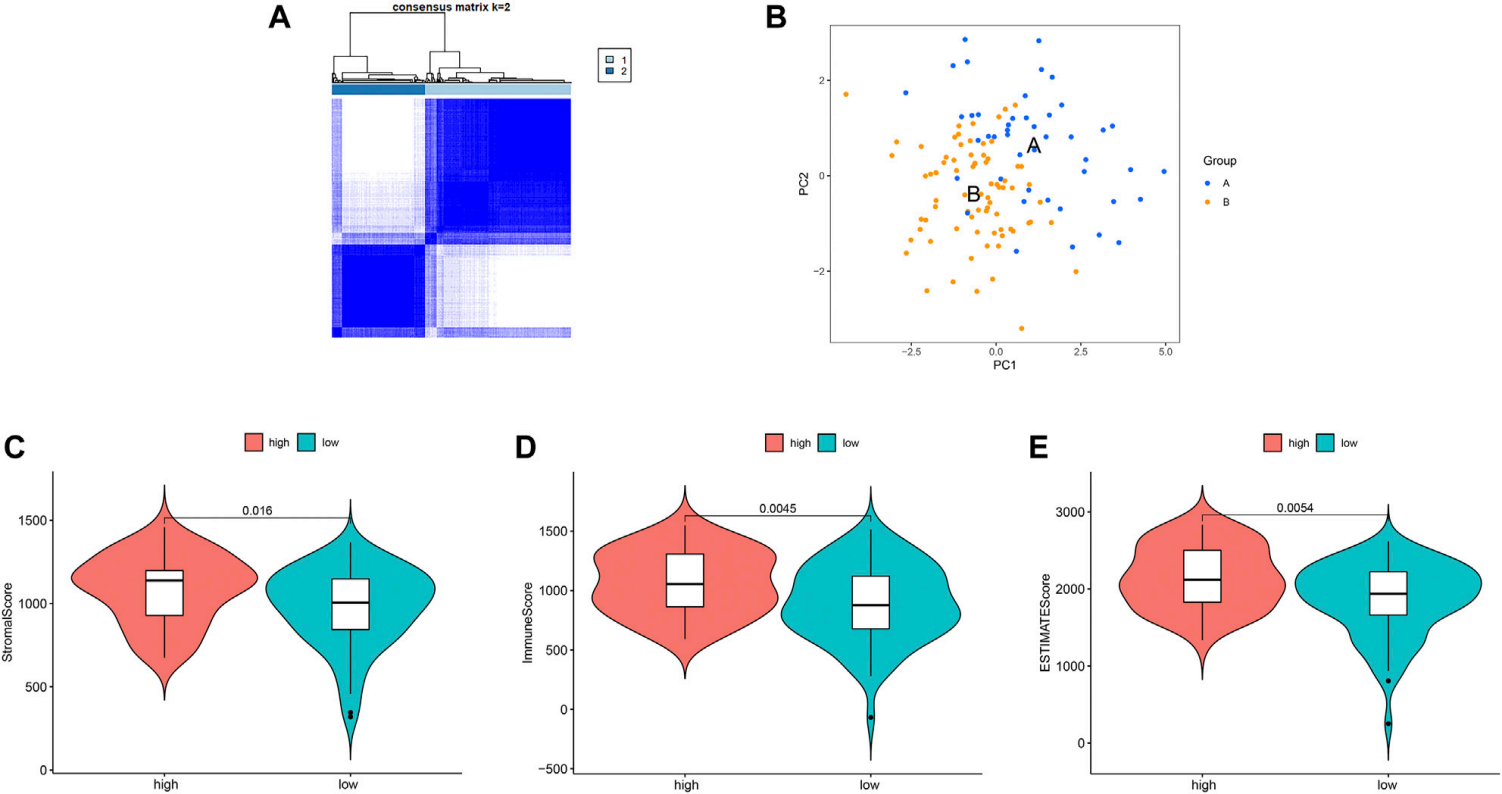
Ferroptosis clusters and correlations with the tumor microenvironment. **(A)** Results obtained from consensus clustering identified two different clusters, namely cluster 1, and 2. **(B)** Two-dimensional plots of two groups with the top two principal components, in which blue dots represented group A, and orange dots represented group B. **(C)** Patients in high-ferroptosis had higher stromal score, immune score and ESTIMATE score, as shown in **(C–E)** respectively.

We identified two different clusters through the consensus cluster analysis in patients with BC (cluster 1: *n* = 45; cluster 2: *n* = 77), and patients in clusters 1 and cluster 2 had different consensus matrices (Figure 7A). To obtain quantitative indicators of cluster 1 and 2, we further probed into the ferroptosis implications by the PCA, and patients were classified into group A and group B (*n* = 45, *n* = 77 respectively) (Figure 7B). Each patient in group A and group B was computed as the sum of individual relevant individual scores. Next, the ferroptosis score was defined as group A subtracts group B. Patients were stratified into high- or low-ferroptosis according to the median of ferroptosis score. Our data showed that patients with high-ferroptosis scores had significantly higher stromal score, immune score and ESTIMATE score compared with those in group B (Figures 7C–E, respectively), indicating the ferroptosis had dramatic influences on TME features.

### Drug Sensitivity Analysis

**FIGURE 8:**
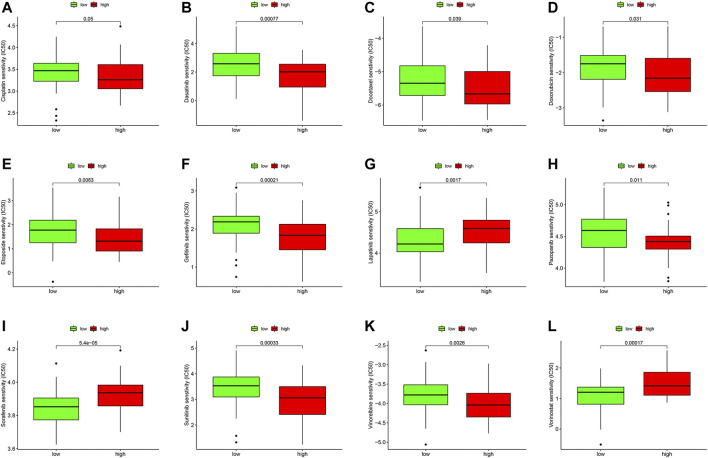
Drug sensitivity analysis between the high- and low-ferroptosis groups. Box plots demonstrated the differences of IC50 in Cisplatin **(A)**, Dasatinib **(B)**, Etoposide **(C)**, Gefitinib **(D)**, Lapatinib **(E)**, Pazopanib **(F)**, Sorafenib **(G)**, Sunitinib **(H)**, Vorinostat **(I)**, Docetaxel **(J)**, Vinorelbine **(K)**, and Doxorubicin **(L)**. The lower the IC50, the more sensitive the drug is.

We compared the IC50 differences of 12 targeted and chemotherapeutic agents, including the Cisplatin (Figure 8A), Dasatinib (Figure 8B), Etoposide (Figure 8C), Gefitinib (Figure 8D), Lapatinib (Figure 8E), Pazopanib (Figure 8F), Sorafenib (Figure 8G), Sunitinib (Figure 8H), Vorinostat (Figure 8I), Docetaxel (Figure 8J), Vinorelbine (Figure 8K), and Doxorubicin (Figure 8L). Our results demonstrated that the IC50 levels of Cisplatin, Dasatinib, Etoposide, Gefitinib, Lapatinib, Pazopanib, Sunitinib, Docetaxel, and Vinorelbine were higher in the low-ferroptosis score group, indicating patients with high-ferroptosis scores were more sensitive to these drugs. Oppositely, the IC50 of Sorafenib, Vorinostat and Doxorubicin was higher in the high-ferroptosis score group, indicating that patients in the low-ferroptosis group were more sensitive to the three drugs.

### Potential Small Molecular Compounds

**FIGURE 9:**
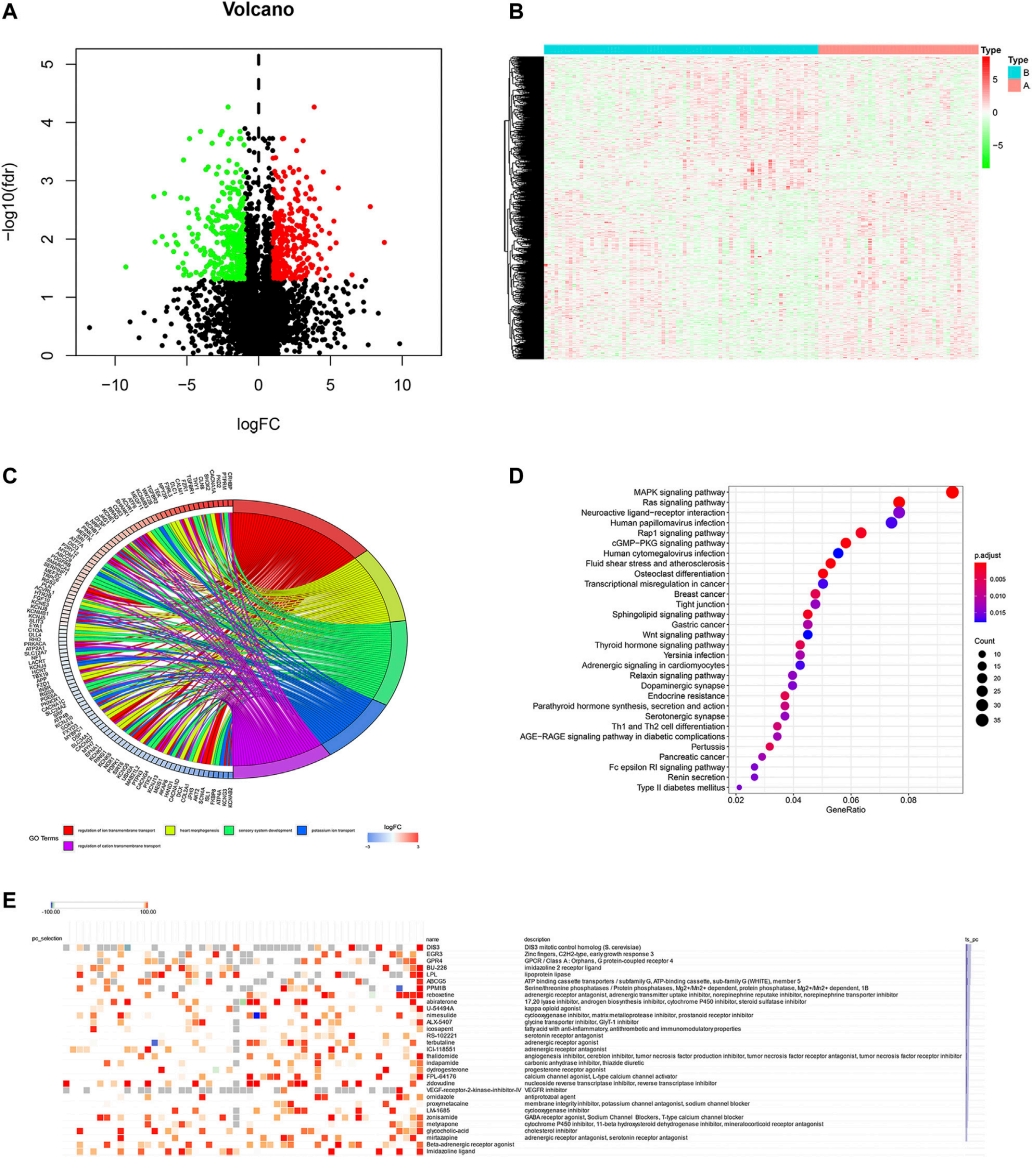
Potential small molecule compounds based on the ferroptosis. **(A)** The volcano plot demonstrated the up-regulated genes (red dots) and down-regulated genes (green dots). **(B)** Heatmap showed the SDG expression profiles between high- and low-ferroptosis score groups. **(C)** GO circle plot showed the enrichment analysis results ranked by logFC and FDR. **(D)** KEGG enrichment analysis revealed the possible pathways and mechanisms. **(E)** Small molecular compounds and possible mechanisms. The results were downloaded from the CMap website.

To screen the potential small molecular compounds that may target patients with BC, we further analyzed the SDG between high- and low-ferroptosis groups. There were 1183 SDG with the FDR <0.05, including the 522 up-regulated and 661 down-regulated genes. The volcano plot and heatmap were visualized in Figures 9A,B. GO enrichment analysis showed the SDG were significantly associated with ion transmembrane transport, potassium and cation transmembrane transport pathways (Figure 9C). KEGG results showed the SDG were strongly associated with MAPK, Ras, and Rap1 signal pathways (Figure 9D). Then, the up- and down-regulated genes were uploaded into the CMap database. A total of 29 small molecular compounds and 30 mechanisms were identified (Figures 9E,F, respectively). The details of the small molecular compounds were provided in Supplementary Table S5.

### Ferroptosis and Other Metastatic Sites in Patients With Breast Cancer

To explore whether ferroptosis has any effects on other metastatic sites of breast cancer, we analyzed the FRG expression profiles in lung metastasis and lymph nodes metastasis in GSE10893. There are four lung metastasis samples, nine lymph nodes samples and 67 breast cancer samples in GPL887 in GSE10893. The results demonstrated that MT1G, SQLE, NQO1, and GOT1 were significantly differently expressed between BC and lung metastasis (all *p* < 0.05). In addition, HSBP1, FTH1, and ACSF2 expression levels were significantly different in lymph nodes metastasis sites compared to BC (all *p* < 0.05). The details can be seen in Supplementary Table S6. These finding suggested that the occurrences of lung metastasis and lymph nodes metastasis are companied by a small amount of aberrant FRG expressions.

### External Validation of FRG and Survival Analysis

**FIGURE 10:**
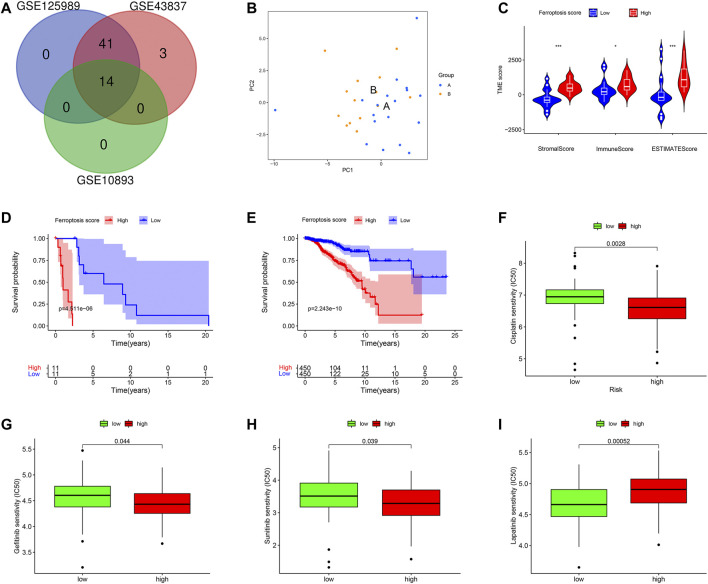
External validations based on ferroptosis. **(A)** The venn graph demonstrated there were 14 intersection FRG among the three datasets. **(B)** Principal component analysis to classify patients with BCBM according to the ferroptosis. **(C)** Patients in high-ferroptosis had higher stromal score, immune score and ESTIMATE score. **(D)** K-M survival plot for patients with high- and low-ferroptosis scores in metastatic group. **(E)** K-M survival plot for patients with high- and low-ferroptosis scores in non-metastatic group. Drug sensitivity analysis of Cisplatin **(F)**, Gefitinib **(G)**, Sunitinib **(H)**, and Lapatinib **(I)**.

We verified the ferroptosis-related genes using another dataset (GSE125989) from GEO, which contained 16 primary BC and 16 matched BCBM tissues. A total of 55 FRG were obtained, and 14 intersection genes were overlapped among the three datasets (GSE10893, GSE43837, and GSE125989) (Figure 10A). Then, to further validate the discriminative abilities of ferroptosis for BC stratification, we performed the PCA analysis and explored the ferroptosis-related cluster patterns and relations with TME. Consistent with the above results, PCA exhibited good ability to classify BCBM patients based on the ferroptosis-related genes expressions (Figure 10B). In addition, Figure 10C demonstrated that patients with high-ferroptosis scores had significantly higher stromal score, immune score and ESTIMATE score in TME, and these results were in line with the above results from GSE10893.

Furthermore, the practicability of the ferroptosis score was validated in TCGA database. Firstly, we classified breast cancer patients into metastasis group and non-metastasis group (*n* = 22, *n* = 906, respectively). Next, we calculated the ferroptosis score for each patient by the method described above, and compared the survival differences between high- and low-ferroptosis score groups. In the metastasis group, the K-M plot showed that patients in the low-ferroptosis group had higher survival probabilities compared to those in high-ferroptosis group (*p* < 0.001) (Figure 10D). Similarly, in the non-metastasis group, patients in the low-ferroptosis score group had better survival than those in the high-ferroptosis score group (*p* < 0.001) (Figure 10E).

In addition, the drug sensitivity was also evaluated in patients from TCGA. In line with the results from GEO, the data demonstrated that patients in the high-ferroptosis score group had lower IC50 of Cisplatin (Figure 10F), Gefitinib (Figure 10G) and Sunitinib (Figure 10H). However, patients in the low-ferroptosis score group had lower IC50 of Lapatinib (Figure 10I), indicating these patients were more sensitive to Lapatinib.

## Discussion

Cell death is of vital importance for normal development, physiological homeostasis and excessive proliferation, such as tumors. Tumor cells exhibit more iron demand than normal cells. Ferroptosis is a newly recognized, iron-dependent form of cell death by Dixon and colleagues in 2012, which shares none of the characteristics of morphology, biochemistry and functions associated with necrosis, apoptosis and autophagy (Dixon et al., 2012). It’s increasingly evident that ferroptosis has been linked to various cancers, especially cancers from iron-rich tissues such as the brain (Zille et al., 2019; Zhang et al., 2021). Studies have demonstrated that altered iron metabolism is correlated closely with the prognosis of breast cancer patients (Shen et al., 2018; Yu et al., 2019a; Nagpal et al., 2019). In addition, ferroptosis could promote tumor metastasis in some cancers (Boult et al., 2008; Ferroptosis Is Inhibited, 2020). Considering the evidence above, it’s reasonable to hypothesize that ferroptosis may be involved in the process of BCBM through an unknown mechanism. Therefore, we explored the roles of ferroptosis in BCBM using large public database by bioinformatics analysis. In this study, we found 14 differently expressed ferroptosis-related genes between the BC and BCBM tissues. Functional enrichment analysis showed these genes were closely associated with the iron ion homeostasis and ferroptosis-related activities. Moreover, TME analysis implied that ferroptosis had intimate crosstalk with immunity and drug sensitivity. Further, survival analysis suggested ferroptosis had prognostic values in predicting patients’ survival.

Despite several studies have investigated the roles of ferroptosis in BC, the underlying mechanisms between ferroptosis and BC cells remain elusive, and the FRG expression profiles of BCBM is never explored (Ma et al., 2016; Yu et al., 2019a; Nagpal et al., 2019). Functional enrichment analysis in this study has demonstrated that ferroptosis may be involved in BCBM through disrupting the iron ion homeostasis. Iron metabolism is tightly associated with iron uptake, utilization, storage and export. High iron level gives rise to reactive oxygen species (ROS) and determines the sensitivity of cells to ferroptosis (Brown et al., 2020; Chen et al., 2020). Subsequently, iron-induced oxidative stress could promote the metastasis initiation possibly through the following mechanisms: 1) modifying the genome, epigenome, leading to the tumor heterogeneity and metastatic abilities; 2) remodeling the extracellular matrix (ECM), which increases the matrix metalloproteinases (MMPs) expression, such as matrix metalloproteinases-9 (MMP-9) that facilitates the metastasis; 3) modulating the tumor microenvironment by restraining the immune response and stimulating the angiogenesis to enable the cancer cell mobility and invasion; 4) changing the metabolic plasticity of cancer cells to compete for and utilize more energy for surviving longer and metastasis; 5) interacting with the secondary site by releasing some signals, such as exosomes to establish a pre-metastatic niche (Kaomongkolgit et al., 2008; Akatsuka et al., 2012; Torti and Torti, 2013a; Torti and Torti, 2013b; Brown et al., 2020). In fact, cancer cells which are prone to metastasis are often highly susceptible to ferroptosis (Hangauer et al., 2017). Consistent with previous findings, our enrichment analysis also showed the iron homeostasis played vital role in the development of breast cancer cells metastasize to the brain. The result highlights the significance of ferroptosis in BCBM and represents a potential approach to prevent metastatic disease.

The FRG signatures proposed in the present study was composed of 14 genes, which could be generally classified into 4 categories, including iron metabolism (ACO1, HMOX1, TFRC), lipid metabolism (ALOX5, CS, LPCAT3, GPX4, PEBP1, FDFT1, PEBP1), (anti)oxidant metabolism (KEAP1, HMOX1) and energy metabolism (GLS2, G6PD) (Stockwell et al., 2017; Hassannia et al., 2019). The correlation network showed the HMOX1 and TFRC were hub genes regulating ferroptosis. HMOX1, also known as HO-1 (heme oxygenase 1), could catalyze the degradation of heme to biliverdin and Fe^2+^. The study showed that HMOX1 knockout could enhance ferroptosis (Adedoyin et al., 2018). The expression of HO-1 is significantly associated with distant metastasis, and predicts an unfavorable OS in patients with BC (Noh et al., 2013). Moreover, clinical correlation analysis showed the HO-1 expression is significant with histologic grade, which was in line with our result (Noh et al., 2013). In contrast to the evidence that the HO-1 expression promotes the ferroptosis (Noh et al., 2013), Li Q et al. demonstrated HO-1 could inhibit mammary tumor metastasis mediated by Notch1 pathway (Li et al., 2019). Hence, the elucidation of HMOX1 in breast cancer metastasis needs to be further investigated. TFRC refers to the transferrin receptor, which promotes ferroptosis by importing iron into cells and sparks ferroptotic cascade reaction ultimately. Notably, TFRC modulates the ROS generation and silencing of TFRC significantly inhibits ferroptosis (Stockwell et al., 2017; Hassannia et al., 2019). Consistent with the previous study, our result also showed the TFRC expression was lower in ER+ BC tissues than that in ER-tissues (Yu et al., 2019b). Therefore, it’s conceivable that the activation degree of ferroptosis is different in ER+ and ER-tissues and this can partly explain why different subtypes of breast cancer have different abilities to metastasize to the brain.

The prognostic model constructed in our study identified KEAP1 and LPCAT3 were independent genes for RFS. Breast cancer patients in high- and low-risk groups exhibit significantly different prognoses (*p* < 0.05), implying risk score based on the FRG signatures has excellent ability to predict survival. Studies about molecular mechanisms revealed KEAP1 could bind to and regulate NRF2 (another ferroptosis-related gene), and its knockdown confers cells’ resistance to ferroptosis (Hassannia et al., 2019). Cumulative evidence demonstrated KEAP1 is associated with worse prognoses in patients with BC, which is concordant with our results (Hartikainen et al., 2015; Almeida et al., 2019). In addition to predicting survival, KEAP1 could also render BC metastasis by interacting with other molecules, for instance, the TrkB and HBXIP (Kim et al., 2016; Zhou et al., 2019). LPCAT3 is a member of the lipid metabolism family, which incorporates acylated fatty acids into membranes and is involved in the biosynthesis of phospholipids (Stockwell et al., 2017; Hassannia et al., 2019). The LPCAT3 knockdown will suppress the ferroptosis (Stockwell et al., 2017). However, the role of LPCAT3 in BC is still in its early stages and much less has been uncovered. So, experimental models *in vitro* and *in vivo* need to be developed to assess the roles of LPCAT3 in BCBM.

Mounting studies from preclinical assays have linked ferroptosis to the immune cells and functions relevant to tumors (Friedmann Angeli et al., 2019; Wang et al., 2019; Tang et al., 2020). Ferroptosis cells will release chemotactic signals, such as lipid mediators to attract antigen-presenting cells (APC) and other immune cells to degrade these aberrant cells (Almeida et al., 2019). It should be stressed that GPX4, an anti-ferroptosis agent, could reduce phospholipid hydroperoxide and repress lipoxygenase-mediated lipid peroxidation (Zhou et al., 2020). Further evidence demonstrated that CD4^+^ and CD8^+^ T cells lacking GPX4 failed to expand and could not prevent immunity to infection (Matsushita et al., 2015). Besides, GPX4 is key factors involved in ferroptosis and lipid metabolism that regulate tumor metastasis (Li and Li, 2020). Similarly, our study also showed that the level of the antigen presentation process and the contents of immune cells were significantly different between high- and low-risk score groups. These studies enforce the notion that ferroptosis will affect immunity and may open up new possibilities to efficiently improve cancer treatment. Several lines of evidence have confirmed that ferroptosis enhanced the tumor suppression mediating by interferon-gamma (INF-γ) secreted by CD8^+^ T cells in response to immune checkpoint blockade (Stockwell and Jiang, 2019; Wang et al., 2019). In turn, immunotherapy-activated T cells also enhance ferroptosis-specific lipid peroxidation in tumor cells (Wang et al., 2019). Immune checkpoint inhibitors have revolutionized the treatment of brain metastasis and breast cancer patients in recent years (Solinas et al., 2017; Lauko et al., 2018). It’s thus tempting to speculate that, if sufficient details between ferroptosis and immunity have been uncovered, patients with BC may benefit more and a shift from anti-tumor to immunosuppressive responses might take place.

There is an increasing trend of clinical trials recruiting patients with BCBM to explore the efficacy of targeted agents. However, the therapeutic response rate is still low, ranging from 6% for alone to 49% for in combination (Pedrosa et al., 2018). Our data suggested that patients in the high-ferroptosis group were more sensitive to Cisplatin, Dasatinib etc., and low-ferroptosis patients were more sensitive to Lapatinib and Sorafenib. This finding could assist the clinicians in selecting suitable patients who may reap survival benefits. In addition, given the limited efficacy of traditional chemotherapy, there is an urgent necessity to exploit new agents that target BC. In this study, we identified 29 molecular compounds, involving the possible mechanisms. Evaluation of the small molecular drugs is crucial to the development of BCBM treatment. A more pronounced understanding of the possible mechanisms through the basic experiments will lead a substantial shift to tailed therapeutic applications (Waks and Winer, 2019).

The strength of our study is that we performed a systematic analysis of FRG signatures in breast cancer patients from national database and validated using external databases, which provided robust statistical support. To our best known, it’s the first time to explore the relationships between ferroptosis and BCBM, which shed light on the significance of ferroptosis in metastatic tumors. It’s also the first time to construct FRG signatures to predict patients’ survival and therapeutic response, which may be useful to assist clinicians in making individualized strategy for patients with BCBM. Meanwhile, there are some limitations in our study. Firstly, this is a retrospective study with data from public repositories. A large-scale and multicenter real-world analysis is warranted to verify these results. Secondly, the mechanisms of how ferroptosis modulates BCBM precisely are still unclear, and metastatic animal models are essential to understand the specific roles of ferroptosis. Lastly, it should be emphasized that the links between breast cancer ferroptosis and immune cells are not fully understood and needs to be validated experimentally. Notwithstanding its limitations, this study does provide a comprehensive overview of FRG profiles in BCBM and these limitations can be solved if there are enough data in the future.

In conclusion, we identified ferroptosis expression profiles that may be involved in the process of BCBM, and the ferroptosis patterns have values in predicting survival and drug sensitivity. New efforts targeting BCBM should incorporate the idea that ferroptosis could influence the breast cancer microenvironment.

## Data Availability

The original contributions presented in the study are included in the article/Supplementary Material, further inquiries can be directed to the corresponding authors.

## Glossary

APC: antigen-presenting cells
AUC: area under curves
BC: breast cancer
BCBM: breast cancer brain metastasis
BP: biological process
CC: cellular component
CI: confidence interval
ECM: extracellular matrix
ER: estrogen receptor
FRG: ferroptosis-related genes
GEO: Gene Expression Omnibus
GO: Gene Ontology
GPX4: glutathione peroxidase 4;
GSDC: Genomics of Drug Sensitivity in Cancer
GSH: glutathione
HO-1: heme oxygenase 1
HR: hazard ratio
INF-γ: interferon gamma
KEGG: Kyoto Encyclopedia of Genes and Genomes
K-M: Kaplan-Meier
MF: molecular function
MMPs: matrix metalloproteinases
MMP-9: matrix metalloproteinases-9
OS: overall survival
PCA: principal component analysis
PgR: progesterone receptor
RFS: relapse-free survival
ROC: receiver operating curves
ROS: reactive oxygen species
SDG: significantly different genes
ssGSEA: single-sample gene set enrichment analysis
TCGA: The Cancer Genome Atlas
TME: tumor microenvironment
TNBC: triple-negative breast cancer.
