# Supplementary figures and images for: Genomic Analysis Uncovers Immune Microenvironment Characteristics and Drug Sensitivity of Ferroptosis in Breast Cancer Brain Metastasis
Source: Front Genet. 2022 Jan 25;12:819632. doi: 10.3389/fgene.2021.819632 (PMC8830939; doi:10.3389/fgene.2021.819632)

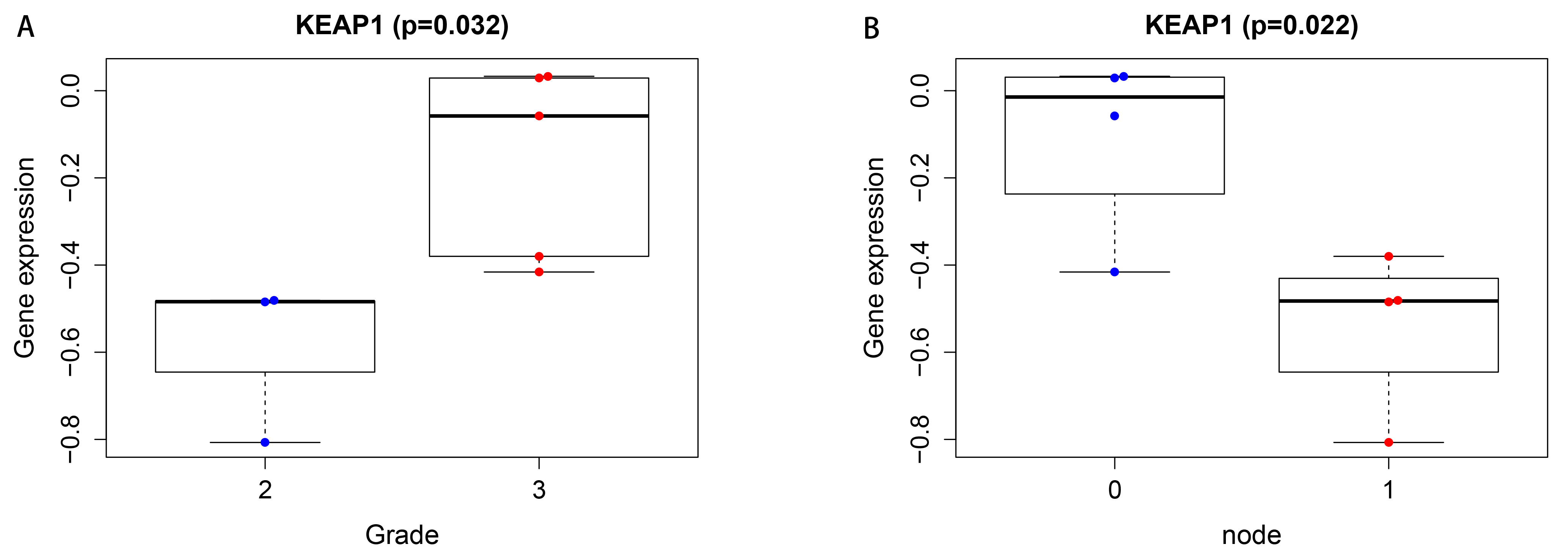

Supplement: Supplementary file 1 [file Image3.TIF]

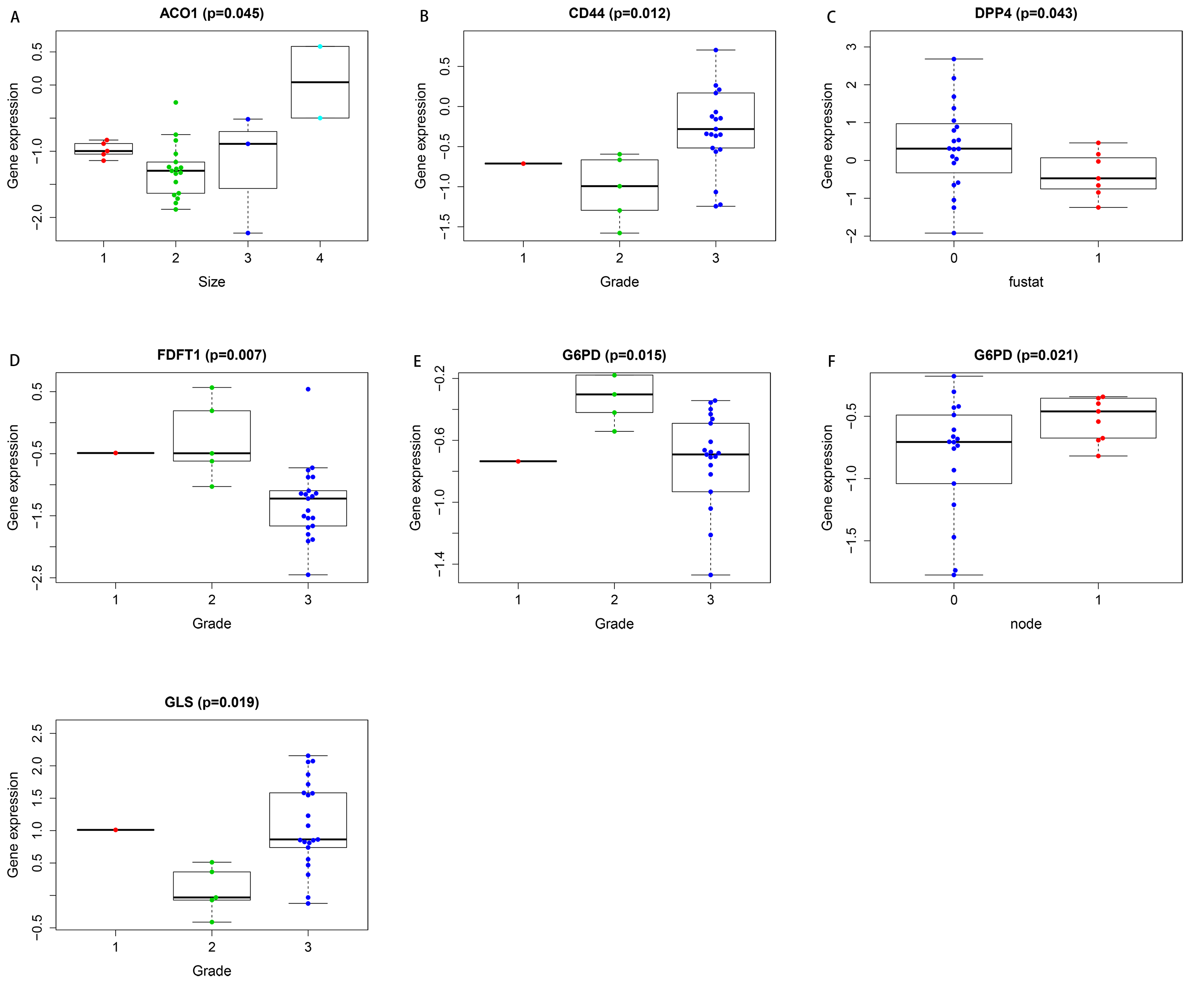

Supplement: Supplementary file 2 [file Image4.TIF]

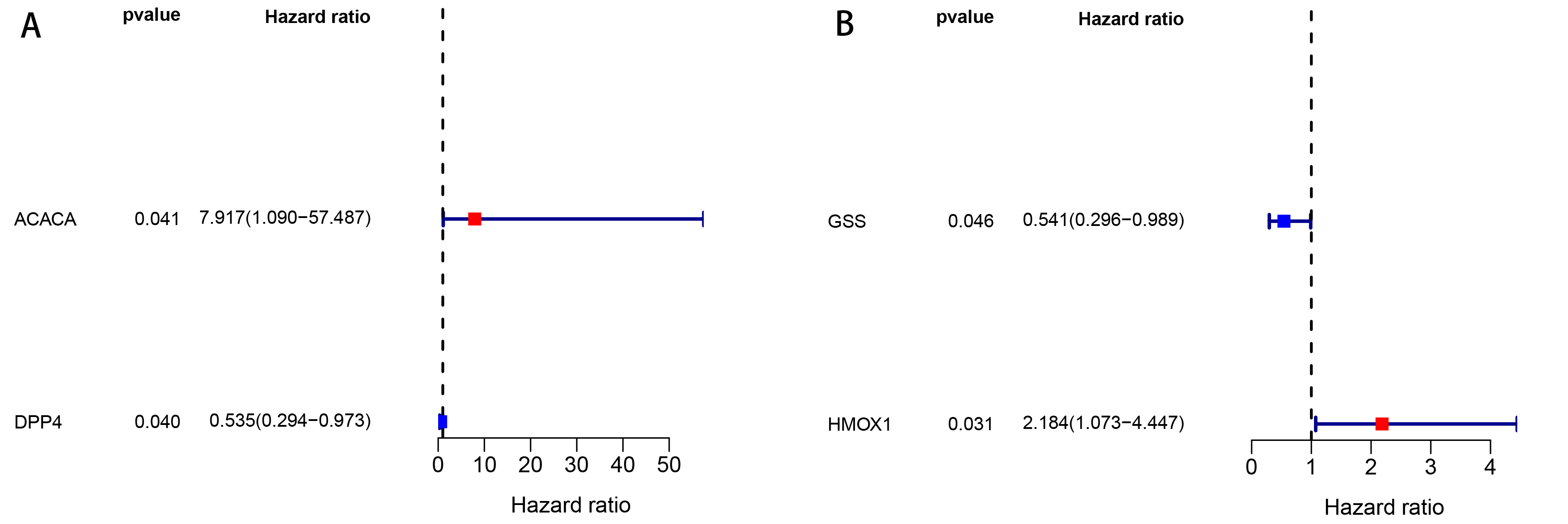

Supplement: Supplementary file 3 [file Image2.TIF]

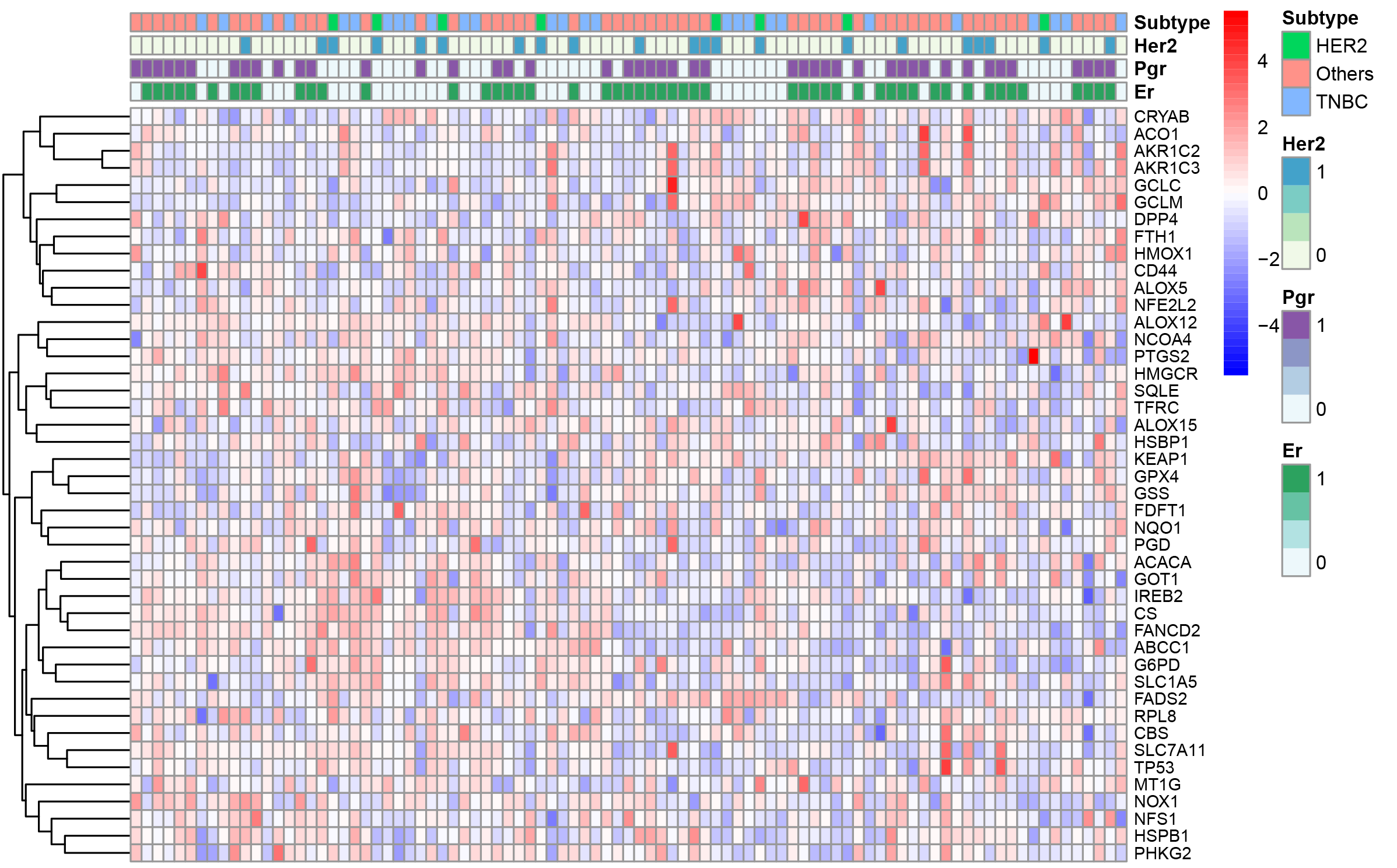

Supplement: Supplementary file 4 [file Image1.TIF]

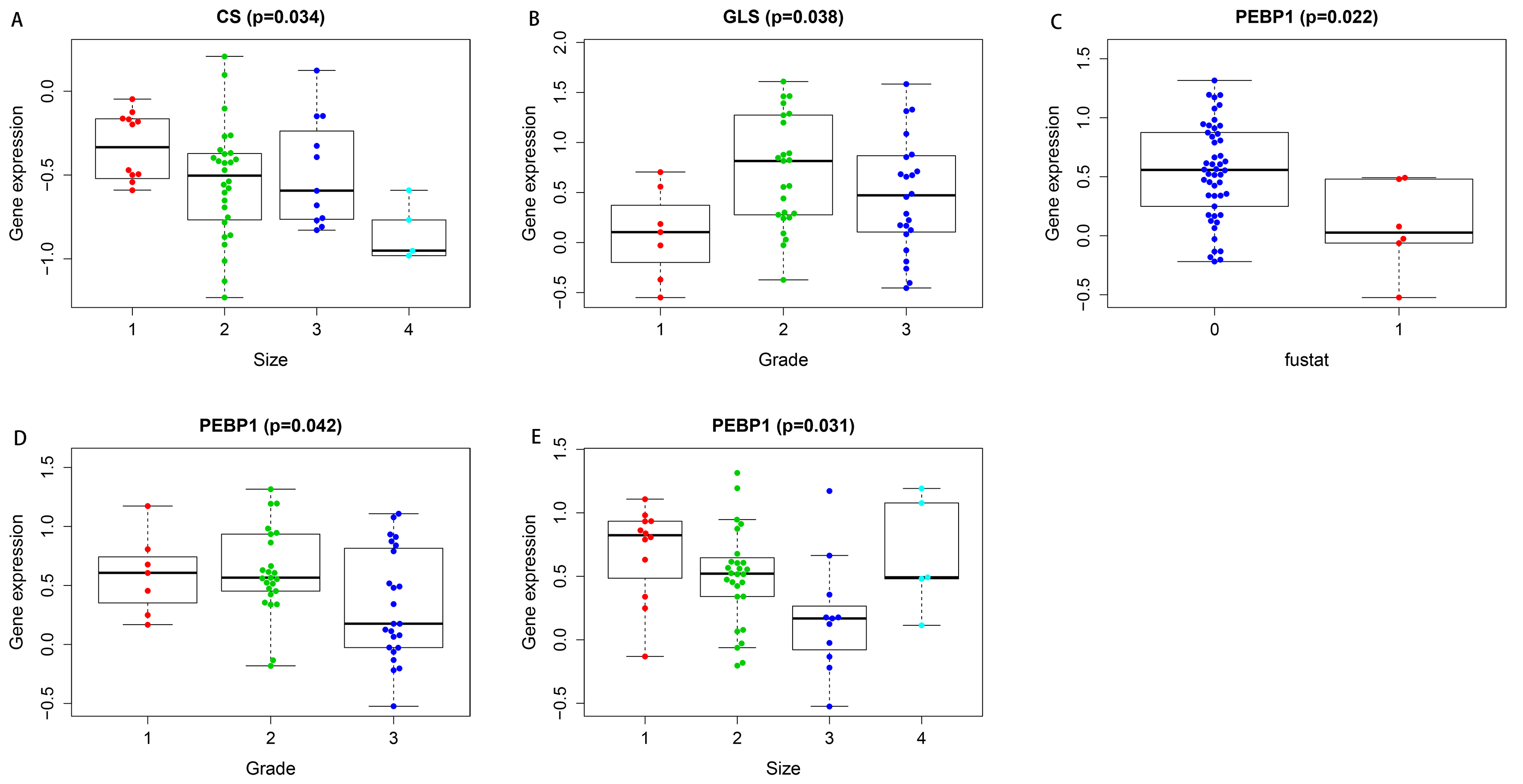

Supplement: Supplementary file 5 [file Image5.TIF]
